# Impact of homocysteine levels on clinical outcome in patients with acute ischemic stroke receiving intravenous thrombolysis therapy

**DOI:** 10.7717/peerj.9474

**Published:** 2020-07-10

**Authors:** Lei Li, Xiaoye Ma, Li Zeng, Sajan Pandey, Ronghao Wan, Rui Shen, Quanbin Zhang

**Affiliations:** 1 Department of Neurosurgery, Shanghai Tenth People’s Hospital, Tongji University School of Medicine, Shanghai, China; 2 Department of Neurology, Shanghai Tenth People’s Hospital, Tongji University School of Medicine, Shanghai, China

**Keywords:** Acute ischemic stroke, Alteplase, Intravenous thrombolysis, Modified Rankin Scale, Homocysteine

## Abstract

**Background:**

The purpose of this study was to retrospectively assess the potential correlation between clinical outcomes and homocysteine (Hcy) levels in acute ischemic stroke (AIS) patients after recombinant tissue plasminogen activator (rtPA) treatment.

**Methods:**

AIS patients treated by rtPA were enrolled between September 2018 and March 2019 in the Stroke Center (Department of Neurology and Neurosurgery), Shanghai Tenth People’s Hospital, Tongji University School of Medicine. Demographics, baseline and clinical characteristics, and modified Rankin Scale (mRS) score after three months from the onset were retrospectively analyzed. Then we compared data about demographics, baseline and clinical characteristics between patients with favorable (mRS score 0–2) and unfavorable (mRS score 3–6) outcomes.

**Results:**

Among 141 patients, 36 patients had poor outcome, for an incidence of 25.53%. Univariate analysis showed that higher Hcy levels (OR = 1.07, 95% CI [1.02–1.12]), older age (OR = 1.06, 95% CI [1.02–1.10]), longer door to needle time (DNT) (OR = 1.03, 95% CI [1.01–1.05]), higher D-Dimer levels (OR = 1.33, 95% CI [1.03–1.71]), and higher National Institutes of Health Stroke Scale (NIHSS) score before treatment (OR = 1.21, 95% CI [1.08–1.35]) were each associated with poor outcome. Also, without internal carotid artery plaque (OR = 0.30, 95% CI [0.10–0.92]) showed a protective effect on patients’ clinical outcome. Patients with higher levels of Hcy decline also showed an increased risk of poor outcome for AIS patients obtaining rtPA treatment (Non-adjusted: OR = 1.07, 95% CI [1.02–1.12]; Adjust model I adjusts for demographics (age, male): OR = 1.06, 95% CI [1.02–1.11]; Adjust model II adjusts for hospital care factors (onset to treatment, DNT): OR = 1.08, 95% CI [1.03–1.13]; Adjust model III adjusts for health and stroke factors (INR, D-Dimer, HGB, NIHSS score before treatment, smoking, drinking, hypertension, diabetes, coronary disease, hyperlipidemia, previous stroke, atrial fibrillation, hemorrhagic transformation, internal carotid artery plaque): OR = 1.06, 95% CI [1.02–1.11]). The results are very stable in all three models constructed.

**Conclusion:**

The results of this study indicate that increased Hcy level independently predicts unfavorable outcome in AIS patients accepting thrombolytic therapy. However, the contribution of Hcy to the outcome, although significant, is relatively small and perhaps not clinically significant when all the other confounders are considered.

## Introduction

Acute ischemic stroke (AIS) is one of the principal causes of disability and death, which causes great economic and mental burden to patients’ families (Merlino et al., 2019; Rosenbaum Halevi et al., 2019). Intravenous thrombolytic therapy and recombinant tissue plasminogen activator (rtPA) have been broadly used in AIS patients within 4.5 h after symptom onset (Valdes Hernandez et al., 2014).

Previous studies have shown that those who have hyperhomocysteinemia (Hhcy) have a higher risk of the development of AIS, and Hhcy is correlated with poor prognosis in AIS patients (Davis Armstrong et al., 2018; Zaric et al., 2019). Besides, the increased risk of cardiovascular disease could also be partially attributed to Hhcy (Borowczyk et al., 2019). Its possible underlying mechanism is that elevated serum homocysteine (Hcy) may lead to endothelial dysfunction (Esse et al., 2019; Wu et al., 2019; Yan et al., 2019), neurotoxicity (Moretti & Caruso, 2019) and up-regulation of thrombosis formation factors (Diao et al., 2019; Jin et al., 2018). However, the likelihood of applying Hcy to prognosticate AIS patients’ clinical outcomes after rtPA therapy hasn’t been thoroughly investigated. Therefore, we designed this retrospective study to assess the potential relationship between clinical outcomes and Hcy in patients with AIS after rtPA therapy.

## Materials and Methods

### Patients

The study was designed as a retrospective study. Patients with AIS taking rtPA treatment were enrolled between September 2018 and March 2019 in a single Stroke Center (Department of Neurology and Neurosurgery, Shanghai Tenth People’s Hospital, Tongji University School of Medicine). The study population included 205 consecutive patients admitted with AIS within 4.5 h of their symptom onset.

### Exclusion criteria

Evidence of severe infection, cardiopulmonary disease, cancer, hepatic or renal disease, or multiple organ dysfunction. Patients with mental disorders, severe cognitive dysfunction, a history of mental problems and abnormal behavior were also excluded.

### Data and design

The institutional ethics committee of Shanghai Tenth People’s Hospital has approved this retrospective study. All investigations and methods were performed in accordance with Shanghai Tenth People’s Hospital’s guidelines and regulations. The consent form was not required due to the retrospective nature and de-identified nature of this retrospective study. The standard cardiological and neurological examinations were performed immediately on the patients’ arrival in the emergency room. Before the initiation of therapy, the information about vital signs, blood chemistry and computed tomography scans was obtained. Information about previous and concomitant diseases were recorded. National Institutes of Health Stroke Scale (NIHSS) score was assessed (Brott et al., 1989). Adverse events of hemorrhage, including symptomatic intracerebral hemorrhage (sICH), were identified.

All patients underwent intravenous (i.v.) thrombolytic therapy using rtPA at a dose of 0.9 mg/kg. And those who were followed up for at least three months and had the available modified Rankin Scale (mRS) score (Van Swieten et al., 1988) at three months from symptom onset were involved in the final retrospective analysis. Then we compared data about demographics, baseline and clinical characteristics between patients with favorable (mRS score 0–2) and unfavorable (mRS score 3–6) outcomes.

### Statistical analysis

Applying the Kolmogorov–Smirnov test to test if the metrological data followed the normal distribution or not. Mean ± standard deviation (SD) was used to express continuous variables that follow the normal distribution, and the median (quartiles) was used to indicate variables that do not follow a normal distribution. Frequencies or percentages were used to indicate categorical variables.

Chi-Square tests and Kruskal–Wallis *H* test or one-way ANOVA test were applied to detect any statistical difference between the means and proportions of the three groups.

Univariate analysis of baseline characteristics and clinical outcome were carried out. In this process, variables were added to the model with recoding into tertiles/binary or without recoding as needed.

Multiple logistic regression models were utilized to assess the association between serum Hcy levels and clinical outcome. Both non-adjusted and multivariate-adjusted models (variables adjusted for demographics (age, male)); hospital care factors (onset to treatment, door to needle time (DNT)); health and stroke factors (INR, D-Dimer, hemoglobin (HGB), NIHSS score before treatment, smoking, drinking, hypertension, diabetes, coronary disease, hyperlipidemia, previous stroke, atrial fibrillation, hemorrhagic transformation, internal carotid artery plaque) were applied. In this step, multivariate logistic regression models with the conditional forward selection method were used to reduce confounding factors’ contribution and evaluate the independent contribution of Hcy to clinical outcome after adjusting different factors. All analyses were performed with the SPSS 22.0. A two-sided significance level of 0.05 was applied to assess statistical significance.

## Results

### General patient characteristics

Among 205 consecutive patients with AIS, 141 (68.78%) patients’ follow-up data could be analyzed. Among them, 36 patients had poor outcomes, for an incidence of 25.53%. The data about baseline clinical findings, demographic characteristics and medical history records were summarized in Table 1.

**Table 1:** Demographic data, baseline clinical findings, medical history and clinical outcome among patients with AIS receiving rtPA treatment.

Hcy tertile	Bottom tertile *n* = 47	Middle tertile *n* = 47	Top tertile *n* = 47	*P*-value
Age, year	64.00 (58.00–69.00)	67.00 (61.00–75.00)	67.00 (60.00–76.00)	0.136
Male, *n* (%)	25 (53.19)	34 (72.34)	34 (72.34)	0.078
Onset to treatment, min	150.00 (110.00–190.00)	154.00 (105.00–188.00)	126.00 (90.00–172.00)	0.103
DNT, min	48.00 (39.00–57.00)	45.00 (40.00–57.00)	45.00 (35.00–53.00)	0.721
INR	0.94 (0.92–1.00)	0.98 (0.94–1.05)	0.97 (0.95–1.02)	0.017
D-Dimer, μg/mL	0.38 (0.21–0.63)	0.68 (0.34–1.76)	0.57 (0.32–1.75)	0.008
HGB, g/dL	5.90 (5.70–7.10)	6.10 (5.70–6.80)	6.10 (5.50–6.40)	0.572
NIHSS score before treatment	5.00 (3.00–6.00)	5.00 (4.00–9.00)	7.00 (4.00–9.00)	0.060
Hcy, μmol/L	7.80 (7.00–9.00)	12.20 (10.90–13.00)	20.50 (16.40–26.10)	<0.001
Smoking, *n* (%)	14 (29.79)	20 (42.55)	21 (44.68)	0.282
Drinking, *n* (%)	8 (17.02)	9 (19.15)	11 (23.40)	0.736
Hypertension, *n* (%)	33 (70.21)	38 (80.85)	38 (80.85)	0.369
Diabetes, *n* (%)	24 (51.06)	19 (40.43)	22 (46.81)	0.587
Coronary disease, *n* (%)	1 (2.13)	4 (8.51)	2 (4.26)	0.354
Hyperlipidemia, *n* (%)	17 (36.17)	18 (38.30)	13 (27.66)	0.521
Previous stroke, *n* (%)	8 (17.02)	7 (14.89)	10 (21.28)	0.716
Atrial fibrillation, *n* (%)	3 (6.38)	11 (23.40)	8 (17.02)	0.072
Hemorrhagic transformation, *n* (%)	3 (6.38)	4 (8.51)	3 (6.38)	0.900
Internal carotid artery plaque, *n* (%)	33 (70.21)	37 (78.72)	36 (76.60)	0.616
mRS at 3 month, *n* (%)				0.096
<3	39 (82.98)	36 (76.60)	30 (63.83)	
>=3	8 (17.02)	11 (23.40)	17 (36.17)	

**Notes:**

*P* value calculated from Kruskal–Wallis *H* test and Chi-Square tests were used to determine any statistical difference between the means and proportions of the three groups.

AIS, acute ischemic stroke; rtPA, recombinant tissue plasminogen activator; Hcy, homocysteine; DNT, door to needle time; INR, international standard ratio; HGB, hemoglobin; NIHSS, national institutes of health stroke scale; mRS, modified rankin scale.

### Univariate logistic regression analysis

Table 2 presents the association between outcomes at 90 days and each baseline characteristic. Consistent with the available literature, higher Hcy levels (OR = 1.07, 95% CI [1.02–1.12]), older age (OR = 1.06, 95% CI [1.02–1.10]), longer DNT (OR = 1.03, 95% CI [1.01–1.05]), higher D-Dimer levels (OR = 1.33, 95% CI [1.03–1.71]), and higher NIHSS score before treatment (OR = 1.21, 95% CI [1.08–1.35]) were each associated with poor outcome. Without internal carotid artery plaque (OR = 0.30, 95% CI [0.10–0.92]) showed a protective effect on patients’ clinical outcome. The OR for poor outcomes was 0.36 (95% CI [0.14–0.95]), 0.24 (95% CI [0.08–0.67]), 0.24 (95% CI [0.08–0.67]), 0.09 (95% CI [0.03–0.34]) in individuals with Hcy levels, age, D-Dimer levels, NIHSS score before treatment in the bottom tertile compared with those in the top tertile (Table 2).

**Table 2:** Univariate analysis of baseline characteristics and clinical outcome.

Exposure	Statistics	Clinical outcome at 3 months	*P* value
Hcy, μmol/L	12.20 (9.00–16.45)	1.07 (1.02, 1.12)	0.006
Hcy tertile			
Bottom tertile	47 (33.33%)	0.36 (0.14, 0.95)	0.039
Middle tertile	47 (33.33%)	0.54 (0.22, 1.33)	0.18
Top tertile	47 (33.33%)	reference	
Gender			
Male	93 (65.96%)	1.04 (0.47, 2.33)	
Female	48 (34.02%)	reference	0.917
Age, year	66.00 (60.00–73.50)	1.06 (1.02, 1.10)	0.006
Age tertile			
Bottom tertile	47 (33.33%)	0.24 (0.08, 0.67)	0.006
Middle tertile	47 (33.33%)	0.55 (0.23, 1.33)	0.187
Top tertile	47 (33.33%)	reference	
Onset to treatment, min	139.00 (100.00–182.5)	1.00 (1.00, 1.01)	0.405
Onset to treatment tertile			
Bottom tertile	47 (33.33%)	0.56 (0.21, 1.46)	0.233
Middle tertile	47 (33.33%)	0.90 (0.37, 2.20)	0.820
Top tertile	47 (33.33%)	reference	
DNT, min	45.00 (37.50–55.00)	1.03 (1.01, 1.05)	0.013
DNT tertile			
Bottom tertile	47 (33.33%)	0.52 (0.21, 1.32)	0.170
Middle tertile	47 (33.33%)	0.52 (0.21, 1.32)	0.170
Top tertile	47 (33.33%)	reference	
INR	0.97 (0.93–1.02)	11.61 (0.32, 416.15)	0.179
INR tertile			
Bottom tertile	47 (33.33%)	0.71 (0.27, 1.82)	0.473
Middle tertile	47 (33.33%)	1.00 (0.41, 2.47)	1.000
Top tertile	47 (33.33%)	reference	0.433
D-Dimer, μg/mL	0.54 (0.31–1.21)	1.33 (1.03, 1.71)	0.027
D-Dimer tertile			
Bottom tertile	48 (33.57%)	0.24 (0.08, 0.67)	0.006
Middle tertile	47 (32.87%)	0.55 (0.23, 1.33)	0.187
Top tertile	48 (33.57%)	reference	
Smoking			
Yes	55 (39.01%)	reference	
No	86 (60.99%)	1.64 (0.73, 3.68)	0.231
Drinking			
Yes	28 (19.86%)	reference	
No	113 (80.14%)	1.33 (0.49, 3.58)	0.579
Hypertension			
Yes	109 (77.30%)	reference	
No	32 (22.70%)	0.47 (0.17, 1.32)	0.150
Diabetes			
Yes	65 (46.10%)	reference	
No	76 (53.90%)	0.70 (0.33, 1.49)	0.353
Coronary disease			
Yes	7 (4.96%)	reference	
No	134 (95.04%)	0.44 (0.09, 2.05)	0.293
Hyperlipidemia			
Yes	48 (34.04%)	reference	
No	93 (65.96%)	1.77 (0.76, 4.16)	0.188
Previous stroke			
Yes	25 (17.73%)	reference	
No	116 (82.27%)	1.85 (0.74, 4.67)	0.190
Atrial fibrillation			
Yes	22 (15.60%)	reference	
No	122 (84.40%)	0.84 (0.28, 2.45)	0.743
Hemorrhagic transformation			
Yes	10 (7.09%)	reference	
No	131 (92.91%)	0.79 (0.19, 3.22)	0.737
Internal carotid artery plaque			
Yes	106 (75.18%)	reference	
No	105 (24.82%)	0.30 (0.10, 0.92)	0.034
HGB, g/dL	6.00 (5.65–6.85)	0.95 (0.75, 1.20)	0.639
HGB tertile			
Bottom tertile	45 (33.33%)	0.79 (0.30,2.06)	0.624
Middle tertile	45 (33.33%)	1.24 (0.50, 3.10)	0.642
Top tertile	45 (33.33%)	reference	
NIHSS score before treatment	5.00 (4.00–8.00)	1.21 (1.08, 1.35)	0.001
NIHSS score before treatment tertile			
Bottom tertile	47 (33.33%)	0.09 (0.03,0.34)	<0.001
Middle tertile	47 (33.33%)	0.52 (0.22, 1.22)	0.133
Top tertile	47 (33.33%)	reference	

**Note:**

AIS, acute ischemic stroke; Hcy, homocysteine; DNT, door to needle time; INR, international standard ratio; HGB, hemoglobin; NIHSS, national institutes of health stroke scale; mRS, modified rankin scale.

### Subgroup analysis

We further explored the role of other covariables on the association between Hcy levels and outcome. The impact of Hcy levels on outcome exhibited a significant difference in DNT subgroups (*P* = 0.037), whereas no difference in the following subgroups: age, gender, onset to treatment, INR, D-Dimer, smoking, drinking, hypertension, diabetes, coronary disease, hyperlipidemia, previous stroke, atrial fibrillation, hemorrhagic transformation, internal carotid artery plaque, HGB and NIHSS score before treatment (all *P* value for interaction is more than 0.05) (Table 3).

**Table 3:** Subgroup analysis of Hcy levels with clinical outcome according to covariates by logistic regression.

Subgroup	OR (95% CI)	*P* value	*P* value for interaction
Age tertile			0.603
Bottom tertile	1.09 [1.00–1.19]	0.055	
Middle tertile	1.05 [0.98–1.13]	0.186	
Top tertile	1.06 [0.97–1.15]	0.239	
Gender			0.385
Male	1.06 [1.01–1.12]	0.024	
Female	1.12 [1.00–1.25]	0.047	
Onset to treatment tertile			0.381
Bottom tertile	1.07 [0.98–1.17]	0.120	
Middle tertile	1.01 [0.93–1.10]	0.812	
Top tertile	1.13 [1.03–1.23]	0.010	
DNT tertile			0.037
Bottom tertile	1.15 [1.04–1.28]	0.008	
Middle tertile	1.08 [1.00–1.18]	0.064	
Top tertile	1.00 [0.92–1.09]	0.923	
INR Tertile			0.485
Bottom tertile	1.09 [0.99–1.21]	0.070	
Middle tertile	1.07 [1.00–1.14]	0.052	
Top tertile	1.04 [0.94–1.15]	0.466	
D-Dimer tertile			0.124
Bottom tertile	1.18 [1.02–1.37]	0.030	
Middle tertile	1.05 [0.98–1.12]	0.204	
Top tertile	1.03 [0.94–1.13]	0.585	
Smoking			0.713
Yes	1.09 [1.00–1.18]	0.048	
No	1.07 [1.00–1.13]	0.035	
Drinking			0.807
Yes	1.08 [0.96–1.23]	0.196	
No	1.07 [1.01–1.12]	0.013	
Hypertension			0.282
Yes	1.05 [0.99–1.10]	0.101	
No	1.16 [0.97–1.38]	0.105	
Diabetes			0.066
Yes	1.13 [1.04–1.23]	0.004	
No	1.03 [0.98–1.09]	0.212	
Coronary disease			0.937
Yes	1.08 [0.81–1.44]	0.595	
No	1.07 [1.02–1.12]	0.007	
Hyperlipidemia			0.886
Yes	1.07 [0.96–1.20]	0.218	
No	1.06 [1.01–1.12]	0.020	
Previous stroke			0.556
Yes	1.04 [0.95–1.14]	0.368	
No	1.08 [1.02–1.14]	0.011	
Atrial fibrillation			0.608
Yes	1.11 [0.95–1.30]	0.188	
No	1.06 [1.01–1.12]	0.012	
Hemorrhagic transformation			0.612
Yes	1.00 [0.77–1.30]	1.000	
No	1.07 [1.02–1.12]	0.005	
Internal carotid artery plaque			0.193
Yes	1.06 [1.01–1.12]	0.019	
No	1.07 [0.98–1.16]	0.133	
HGB tertile			0.506
Bottom tertile	1.07 [0.99–1.16]	0.085	
Middle tertile	1.05 [0.98–1.11]	0.146	
Top tertile	1.14[1.01–1.28]	0.032	
NIHSS score before treatment tertile			0.791
Bottom tertile	0.83 [0.60–1.16]	0.275	
Middle tertile	1.21 [1.06–1.38]	0.005	
Top tertile	1.03 [0.97–1.09]	0.383	

**Note:**

Hcy, homocysteine; DNT, door to needle time; INR, international standard ratio; HGB, hemoglobin; NIHSS, national institutes of health stroke scale; mRS, modified rankin scale.

### Multivariate logistic regression analysis

Patients with higher levels of Hcy decline also show an elevated risk of poor outcome for AIS patients obtaining rtPA treatment (Non-adjusted: OR = 1.07, 95% CI [1.02–1.12]; Adjust model I adjusts for demographics (age, male): OR = 1.06, 95% CI [1.02–1.11]; Adjust model II adjusts for hospital care factors (onset to treatment, DNT): OR = 1.08, 95% CI [1.03–1.13]); Adjust model III adjusts for health and stroke factors (INR, D-Dimer, HGB, NIHSS score before treatment, smoking, drinking, hypertension, diabetes, coronary disease, hyperlipidemia, previous stroke, atrial fibrillation, hemorrhagic transformation, internal carotid artery plaque): OR = 1.06, 95% CI [1.02–1.11]. The results are very stable in all three models constructed (Table 4).

**Table 4:** Odds ratios of Hcy levels for the clinical outcome by logistic regression.

Exposure	Non-adjusted OR (95% CI)	*P* value	Adjust Model I OR (95% CI)	*P* value	Adjust Model II OR (95% CI)	*P* value	Adjust Model II OR (95% CI)	*P* value
Hcy	1.07 [1.02–1.12]	0.006	1.06 [1.02–1.11]	0.006	1.08 [1.03–1.13]	0.003	1.06 [1.02–1.11]	0.009

**Notes:**

The non-adjusted model adjusts for none.

Adjust model I adjust for: demographics (age, male).

Adjust model II adjust for: hospital care factors (onset to treatment, DNT).

Adjust model III adjust for: health and stroke factors (INR, D-Dimer, HGB, NIHSS score before treatment, smoking, drinking, hypertension, diabetes, coronary disease, hyperlipidemia, previous stroke, atrial fibrillation, hemorrhagic transformation, internal carotid artery plaque).

## Discussion

Currently, intravenous thrombolysis (IVT) with alteplase is the standard treatment approved for AIS patients within 4.5 h of the onset of their symptoms (Valdes Hernandez et al., 2014). In this study, we found that high level of Hcy, as a predictor for poor prognosis at three months in AIS patients receiving rtPA therapy, has important clinical implications. We also demonstrated that the risk of poor prognosis in AIS patients taking IVT increased by approximately 10% for every 1 μmol/L increase in serum Hcy concentration.

In the past decade, an enormous amount of epidemiological evidence supports the correlation between high Hcy levels and increased risk of the development of AIS (Cheng et al., 2018; Lu et al., 2018b), and some researchers have discovered that increased serum Hcy levels are related to increased hematoma volume (Hacke et al., 2008; Zhou et al., 2015). Previous studies also demonstrated that increased Hcy levels are correlated with functional disability in the acute phase of stroke (Mizrahi et al., 2005; Song et al., 2009). A prospective multicenter research conducted by Kwon et al. unveiled that the risk of early neurological deterioration increased along with the increase of Hcy levels in patients with ischemic stroke (Kwon et al., 2014). However, whether high Hcy levels can be regarded as an independent risk factor for unfavorable clinical outcome in ischemic stroke patients accepting IVT has not been well addressed. Though a small number of studies have already been published, the prognostic value of Hcy levels in patients with AIS after IVT remains controversial. Some investigations revealed that Hhcy was correlated with unfavorable outcomes in patients with ischemic stroke (Ling et al., 2018; Luo et al., 2019; Yao et al., 2016), whereas some other studies indicated that there was no significant correlation between Hhcy and ischemic stroke patients’ clinical outcome (Ribo et al., 2004a, 2004b). To further confirm the association between Hcy levels and clinical outcome of AIS patients treated with thrombolytic therapy, we conducted this retrospective study. Consistent with the previous retrospective studies conducted by Ling et al. (2018), Luo et al. (2019) and Yao et al. (2016), we found that there exists a correlation between Hcy levels and poor prognosis after acute thrombolytic therapy in AIS patients in this retrospective study. And we indicated that higher Hcy levels have a negative impact on prognosis. The result of our study implements new proof that Hhcy has a negative impact on ischemic stroke patients’ clinical outcome.

The underlying mechanism may be due to impaired vascular wall integrity and disturbance of cerebrovascular permeability resulting from increased levels of Hcy, which may lead to endothelial dysfunction, damage to elastic structures and damage to the basal layer of cerebral arterioles and microvessels (Fan et al., 2017; Mach et al., 1997).

Some studies have consistently shown that hyperhomocysteinemia is an independent risk factor for atherosclerosis’ development, suggesting that raised plasma levels of Hcy are relevant to endothelial dysfunction (Borowczyk et al., 2019; Lu et al., 2018a; Wang et al., 2017). Besides, high Hcy levels also increase low-density lipoproteins oxidation, and the dominant mechanism by which Hcy adversely affects vascular endothelial function involves oxidative stress and bioactive nitric oxide consumption (Miyazaki et al., 2014; Seo et al., 2010).

Our research also has some restrictions. First, this study is a single-center retrospective study and the sample size is limited. Second, we did not include patients who did not receive thrombolytic therapy, thus may resulting in a selective bias. Third, the Hcy level could be affected by various factors, such as genetic factors and drugs. But we didn’t evaluate the reason for Hhcy in this study cohort. Also, almost all ORs for Hcy are close to 1 in our results. The contribution of Hcy to the outcome, although significant, is relatively small and perhaps not clinically significant when considering all other confounders. Hence, the results should be explained with caution, and the results should be further confirmed in a multicenter prospective study with a larger cohort to clearly establish the correlation between Hhcy and unfavorable outcome in ischemic stroke patients accepting IVT.

## Conclusions

In conclusion, the results of this study indicate that increased Hcy level independently predicts unfavorable outcome in AIS patients accepting thrombolytic therapy. However, the contribution of Hcy to the outcome, although significant, is relatively small when all the other confounders are considered. To better guide clinical practice, the further multicenter prospective study still needs to be done to clearly clarify the correlation between Hcy level and clinical outcome of AIS patients treating with intravenous thrombolysis.

## Supplemental Information

10.7717/peerj.9474/supp-1 Supplemental Information 1 Raw data.

## Additional Information and Declarations

### Data Availability

The following information was supplied regarding data availability: The raw data are available in Table S1.

## References

[ref-1] Borowczyk K, Piechocka J, Glowacki R, Dhar I, Midtun O, Tell GS, Ueland PM, Nygard O, Jakubowski H (2019). Urinary excretion of homocysteine thiolactone and the risk of acute myocardial infarction in coronary artery disease patients: the WENBIT trial. Journal of Internal Medicine.

[ref-2] Brott T, Adams HP, Olinger CP, Marler JR, Barsan WG, Biller J, Spilker J, Holleran R, Eberle R, Hertzberg VJS (1989). Measurements of acute cerebral infarction: a clinical examination scale. Stroke.

[ref-3] Cheng L-S, Tu W-J, Shen Y, Zhang L-J, Ji K (2018). Combination of high-sensitivity C-reactive protein and homocysteine predicts the post-stroke depression in patients with ischemic stroke. Molecular Neurobiology.

[ref-4] Davis Armstrong NM, Chen W-M, Brewer MS, Williams SR, Sale MM, Worrall BB, Keene KL (2018). Epigenome-wide analyses identify two novel associations with recurrent stroke in the vitamin intervention for stroke prevention clinical trial. Frontiers in Genetics.

[ref-5] Diao L, Bai L, Jiang X, Li J, Zhang Q (2019). Long‐chain noncoding RNA GAS5 mediates oxidative stress in cardiac microvascular endothelial cells injury. Journal of Cellular Physiology.

[ref-6] Esse R, Barroso M, De Almeida IT, Castro R (2019). The contribution of homocysteine metabolism disruption to endothelial dysfunction: state-of-the-art. International Journal of Molecular Sciences.

[ref-7] Fan CD, Sun JY, Fu XT, Hou YJ, Li Y, Yang MF, Fu XY, Sun BL (2017). Astaxanthin attenuates homocysteine-induced cardiotoxicity in vitro and in vivo by inhibiting mitochondrial dysfunction and oxidative damage. Frontiers in Physiology.

[ref-8] Hacke W, Kaste M, Bluhmki E, Brozman M, Davalos A, Guidetti D, Larrue V, Lees KR, Medeghri Z, Machnig T, Schneider D, Von Kummer R, Wahlgren N, Toni D (2008). Thrombolysis with alteplase 3 to 4.5 hours after acute ischemic stroke. New England Journal of Medicine.

[ref-9] Jin P, Bian Y, Wang K, Cong G, Yan R, Sha Y, Ma X, Zhou J, Yuan Z, Jia S (2018). Homocysteine accelerates atherosclerosis via inhibiting LXRα–mediated ABCA1/ABCG1–dependent cholesterol efflux from macrophages. Life Sciences.

[ref-10] Kwon HM, Lee YS, Bae HJ, Kang DW (2014). Homocysteine as a predictor of early neurological deterioration in acute ischemic stroke. Stroke.

[ref-11] Ling C, Hong Z, Yu W, Yu L, Xin S (2018). The serum homocysteine level in patients with acute ischemic stroke (AIS) after thrombolysis and its relationship with clinical outcomes. Revista da Associação Médica Brasileira.

[ref-12] Lu S, Deng J, Liu H, Liu B, Yang J, Miao Y, Li J, Wang N, Jiang C, Xu Q, Wang X, Feng J (2018a). PKM2-dependent metabolic reprogramming in CD4+ T cells is crucial for hyperhomocysteinemia-accelerated atherosclerosis. Journal of Molecular Medicine.

[ref-13] Lu SS, Xie J, Su CQ, Ge S, Shi HB, Hong XN (2018b). Plasma homocysteine levels and intracranial plaque characteristics: association and clinical relevance in ischemic stroke. BMC Neurology.

[ref-14] Luo Y, Jin H, Guo ZN, Zhang P, Zhang LY, Chen J, Yu Y, Wang Y, Liu J, He QY, Sun X, Yang Y (2019). Effect of hyperhomocysteinemia on clinical outcome and hemorrhagic transformation after thrombolysis in ischemic stroke patients. Frontiers in Neurology.

[ref-15] Mach F, Schonbeck U, Bonnefoy JY, Pober JS, Libby P (1997). Activation of monocyte/macrophage functions related to acute atheroma complication by ligation of CD40: induction of collagenase, stromelysin, and tissue factor. Circulation.

[ref-16] Merlino G, Corazza E, Lorenzut S, Gigli G, Cargnelutti D, Valente M (2019). Efficacy and safety of intravenous thrombolysis in patients with acute ischemic stroke and pre–existing disability. Journal of Clinical Medicine.

[ref-17] Miyazaki A, Sagae N, Usami Y, Sato M, Kameda T, Yoshimoto A, Ishimine N, Matsuda K, Sugano M, Hara M, Honda T, Tozuka M (2014). N-homocysteinylation of apolipoprotein A-I impairs the protein’s antioxidant ability but not its cholesterol efflux capacity. Biological Chemistry.

[ref-18] Mizrahi EH, Fleissig Y, Arad M, Adunsky A (2005). Plasma homocysteine level and functional outcome of patients with ischemic stroke. Archives of Physical Medicine and Rehabilitation.

[ref-19] Moretti R, Caruso P (2019). The controversial role of homocysteine in neurology: from labs to clinical practice. International Journal of Molecular Sciences.

[ref-20] Ribo M, Montaner J, Molina CA, Arenillas JF, Santamarina E, Alvarez-Sabín J (2004a). Admission fibrinolytic profile predicts clot lysis resistance in stroke patients treated with tissue plasminogen activator. Thrombosis and Haemostasis.

[ref-21] Ribo M, Montaner J, Molina CA, Arenillas JF, Santamarina E, Quintana M, Alvarez-Sabín J (2004b). Admission fibrinolytic profile is associated with symptomatic hemorrhagic transformation in stroke patients treated with tissue plasminogen activator. Stroke.

[ref-22] Rosenbaum Halevi D, Bursaw AW, Karamchandani RR, Alderman SE, Breier JI, Vahidy FS, Aden JK, Cai C, Zhang X, Savitz SI (2019). Cognitive deficits in acute mild ischemic stroke and TIA and effects of rt-PA. Annals of Clinical and Translational Neurology.

[ref-23] Seo H, Oh H, Park H, Park M, Jang Y, Lee M (2010). Contribution of dietary intakes of antioxidants to homocysteine-induced low density lipoprotein (LDL) oxidation in atherosclerotic patients. Yonsei Medical Journal.

[ref-24] Song IU, Kim JS, Ryu SY, Lee SB, Lee SJ, Jeong DS, Kim YI, Lee KS (2009). Are plasma homocysteine levels related to neurological severity and functional outcome after ischemic stroke in the Korean population?. Journal of the Neurological Sciences.

[ref-25] Valdes Hernandez MC, Piper RJ, Bastin ME, Royle NA, Maniega SM, Aribisala BS, Murray C, Deary IJ, Wardlaw JM (2014). Morphologic, distributional, volumetric, and intensity characterization of periventricular hyperintensities. AJNR American Journal of Neuroradiology.

[ref-26] Van Swieten JC, Koudstaal PJ, Visser MC, Schouten HJ, Van Gijn J (1988). Interobserver agreement for the assessment of handicap in stroke patients. Stroke.

[ref-27] Wang R, Wang Y, Mu N, Lou X, Li W, Chen Y, Fan D, Tan H (2017). Activation of NLRP3 inflammasomes contributes to hyperhomocysteinemia-aggravated inflammation and atherosclerosis in apoE-deficient mice. Laboratory Investigation.

[ref-28] Wu X, Zhang L, Miao Y, Yang J, Wang X, Wang CC, Feng J, Wang L (2019). Homocysteine causes vascular endothelial dysfunction by disrupting endoplasmic reticulum redox homeostasis. Redox Biology.

[ref-29] Yan P, Sun C, Ma J, Jin Z, Guo R, Yang B (2019). MicroRNA-128 confers protection against cardiac microvascular endothelial cell injury in coronary heart disease via negative regulation of IRS1. Journal of Cellular Physiology.

[ref-30] Yao ES, Tang Y, Xie MJ, Wang MH, Wang H, Luo X (2016). Elevated homocysteine level related to poor outcome after thrombolysis in acute ischemic stroke. Medical Science Monitor.

[ref-31] Zaric BL, Obradovic M, Bajic V, Haidara MA, Jovanovic M, Isenovic ER (2019). Homocysteine and hyperhomocysteinaemia. Current Medicinal Chemistry.

[ref-32] Zhou F, Chen B, Chen C, Huang J, Chen S, Guo F, Hu Z (2015). Elevated homocysteine levels contribute to larger hematoma volume in patients with intracerebral hemorrhage. Journal of Stroke and Cerebrovascular Diseases.

